# Single-Tube Reverse Transcription–Loop-Mediated Isothermal Amplification Assay for Rapid Detection of Red-Spotted Grouper Nervous Necrosis Virus in Fish Species

**DOI:** 10.3390/v18080827

**Published:** 2026-07-27

**Authors:** Mangottil Ayyappan Pradeep, Cherammpillil Sukumaran Subin, Gokhlesh Kumar, Sulumane Ramachandra Krupesha Sharma, Nadiyath Karayi Sanil, Thaliyil Veetil Arun Kumar, Nikathil Raveendranathan Dhanutha, Thevanattil Sairanksha Azhar Shahansha, Koyadan Kizhakkedath Vijayan

**Affiliations:** Marine Biotechnology, Fish Nutrition and Health Division, Indian Council of Agricultural Research—Central Marine Fisheries Research Institute, Kochi 682018, India

**Keywords:** Nodaviridae, viral nervous necrosis, viral encephalopathy and retinopathy, red-spotted grouper nervous necrosis virus, barfin flounder nervous necrosis virus

## Abstract

Betanodavirus is a causative agent of viral nervous necrosis (VNN) and a major threat to marine and brackish-water aquaculture globally. This virus causes epizootic outbreaks with particularly high morbidity and mortality in larval and juvenile stages and causes significant economic losses in aquaculture. Here, we developed a rapid and highly sensitive single-tube Reverse Transcription–Loop-Mediated Isothermal Amplification (RT-LAMP) assay for the detection of red-spotted grouper nervous necrosis virus (RGNNV) genotype infection in fish tissue samples. Six primers targeting eight conserved regions of the RNA2 coat protein gene of RGNNV were designed with conservation regions across RGNNV genotypes. RT-LAMP assay was completed within 60 min at 65 °C using a single-tube format that combined reverse transcription and isothermal amplification, and results were directly visualized by the addition of SYBR Green I dye, producing a colour change from orange (negative) to green (positive), observable with the naked eye or under UV illumination. The developed RT-LAMP assay was able to detect five copies of RGNNV from infected samples, which was 20-fold more sensitive than conventional reverse transcription-PCR. The assay demonstrated diagnostic sensitivity and specificity in two fish hosts (Asian seabass and cobia) and showed no cross reactivity with other fish viruses such as tilapia lake virus and cyprinid herpesvirus-2. The developed assay is simple, cost-effective, specific, and enables rapid detection of RGNNV in fish tissues. This single-tube RT-LAMP assay can be applied to screening broodstock facilities, fingerlings, aquaculture farms, quarantine facilities, and juveniles before stocking in ponds or cages, helping prevent disease outbreaks and the transmission of RGNNV in aquaculture systems.

## 1. Introduction

Viral nervous necrosis (VNN) or viral encephalopathy and retinopathy (VER) is a serious disease affecting larval and juvenile marine fishes, causing severe economic losses to the aquaculture industry worldwide. The disease affects more than 120 species, mainly of marine origin with reports of occurrences in several freshwater species, suggesting ongoing viral adaptation to novel hosts [1,2]. The virus has a wide geographical distribution and has been reported from all continents [2,3]. VNN is caused by non-enveloped and spherical viruses called betanodaviruses. These are RNA viruses with two molecules of single-stranded RNA. RNA1 (~3.1 kb) encodes the RNA-dependent RNA polymerase essential for viral replication, while RNA2 (~1.4 kb) encodes the major capsid protein, which serves as the primary antigenic target for serological and molecular diagnostics [4,5]. Betanodaviruses are classified into four genotypes based on the RNA2 partial sequence designated striped jack nervous necrosis virus (SJNNV), tiger puffer nervous necrosis virus (TPNNV), barfin flounder nervous necrosis virus (BFNNV), and red-spotted grouper nervous necrosis virus (RGNNV) [6]. Of these, RGNNV exhibits the broadest host range and geographic distribution, and naturally occurring RGNNV/SJNNV reassortant strains have been increasingly identified in European aquaculture systems [5].

Mostly VNN infections occur in juvenile stages and cause massive mortality but infections causing significant mortality in adult fishes have been reported in European sea bass (*Dicentrarchus labrax*), sevenband grouper (*Epinephelus septemfasciatus*), Atlantic halibut (*Hippoglossus hippoglossus*) and golden pompano (*Trachinotus blochii*) [5,7,8,9]. Experimental infections show that other marine fishes are also susceptible to VNN infection, which indicates the possibility of the emergence of a new host. The infected fish that survive are carriers to spread the virus horizontally and vertically [10]. Career adults under severe stress may develop an acute disease. In India, mortality associated with nodavirus infection in hatchery-produced larvae of Asian seabass (*Lates calcarifer*) was first reported by Azad et al. [11]. Betanodavirus was reported from Asian seabass juveniles reared in a brackish water farm [12] and also from Asian seabass juveniles reared in fresh-water cages [13]. This disease has also been reported in other fish species such as cobia (*Rachycentron canadum*) [14], and it has been confirmed that the circulating genotypes in Indian waters predominantly belong to the RGNNV genotype [15].

Conventional diagnostic methods such as histopathology, immunostaining, and cell-culture-based techniques are useful for qualitative diagnosis of betanodavirus; however, they are time-consuming, labour-intensive, often limited to clinically infected cases, and require well-equipped laboratories [16,17]. Furthermore, molecular methods like reverse transcription polymerase chain reaction (RT-PCR) and nested PCR have increased the diagnostic sensitivity and speed of pathogen detection. However, these techniques require expensive instrumentation, trained staff, and are not ideal for on-site detection of betanodavirus.

To overcome these diagnostic limitations, reverse transcription loop-mediated isothermal amplification (RT-LAMP) has emerged as a promising alternative, offering rapid nucleic acid amplification under isothermal conditions amenable to point-of-care deployment [18]. The LAMP technique employs four to six specifically designed primers, including outer (F3/B3), inner (FIP/BIP), and optional loop (LF/LB) primers that recognize six to eight distinct target regions, enabling highly specific amplification through a strand-displacement mechanism under isothermal conditions (60–65 °C), typically yielding detectable amplification within 30–60 min [18,19]. Amplification products can be detected visually through turbidity generated by magnesium pyrophosphate precipitation or by the addition of intercalating fluorescent dyes such as SYBR Green I or calcein, enabling naked-eye result interpretation without specialized instrumentation [19,20]. Compared to PCR-based methods, LAMP reactions demonstrate greater tolerance to common biological inhibitors present in tissue samples, including haemoglobin, bile salts, and mucopolysaccharides, and are typically completed within 60 min [21].

Some RT-LAMP assays have previously been developed for the detection of betanodavirus. Early studies demonstrated that RT-LAMP could detect viral RNA with sensitivity comparable to, or greater than, conventional reverse transcription PCR while substantially reducing the time required for diagnosis [22,23,24,25]. These assays tested the feasibility of RT-LAMP for the rapid diagnosis of VNN in aquatic organisms. However, most previously reported assays were developed using primer sets designed from geographically restricted virus isolates or individual betanodavirus genotypes and were validated using a limited number of host species. Furthermore, Mekata et al. [26] developed a real-time RT-LAMP assay to detect Japanese RGNNV strain in grouper tissues using a Loopamp real-time turbidimeter. A limitation of this assay is that it requires a dedicated instrument for real-time turbidity monitoring of amplified products, which increases equipment costs and limits its accessibility in resource-limited diagnostic laboratories compared with RT-LAMP assays that use simple heating devices and visual colorimetric detection.

In the present study, we developed a rapid, sensitive, specific, and cost-effective single-tube RT-LAMP for the detection of RGNNV genotype in fish tissues using SYBR Green I fluorescence signal as the visual detection method. To our knowledge, this represents one of the first reports of a single-tube RT-LAMP assay for RGNNV that integrates reverse transcription and amplification in a closed reaction and is evaluated across more than one host species under laboratory conditions.

## 2. Materials and Methods

### 2.1. Fish Sampling, Tissue Sample Preparation and Total RNA Extraction

**Figure 1:**
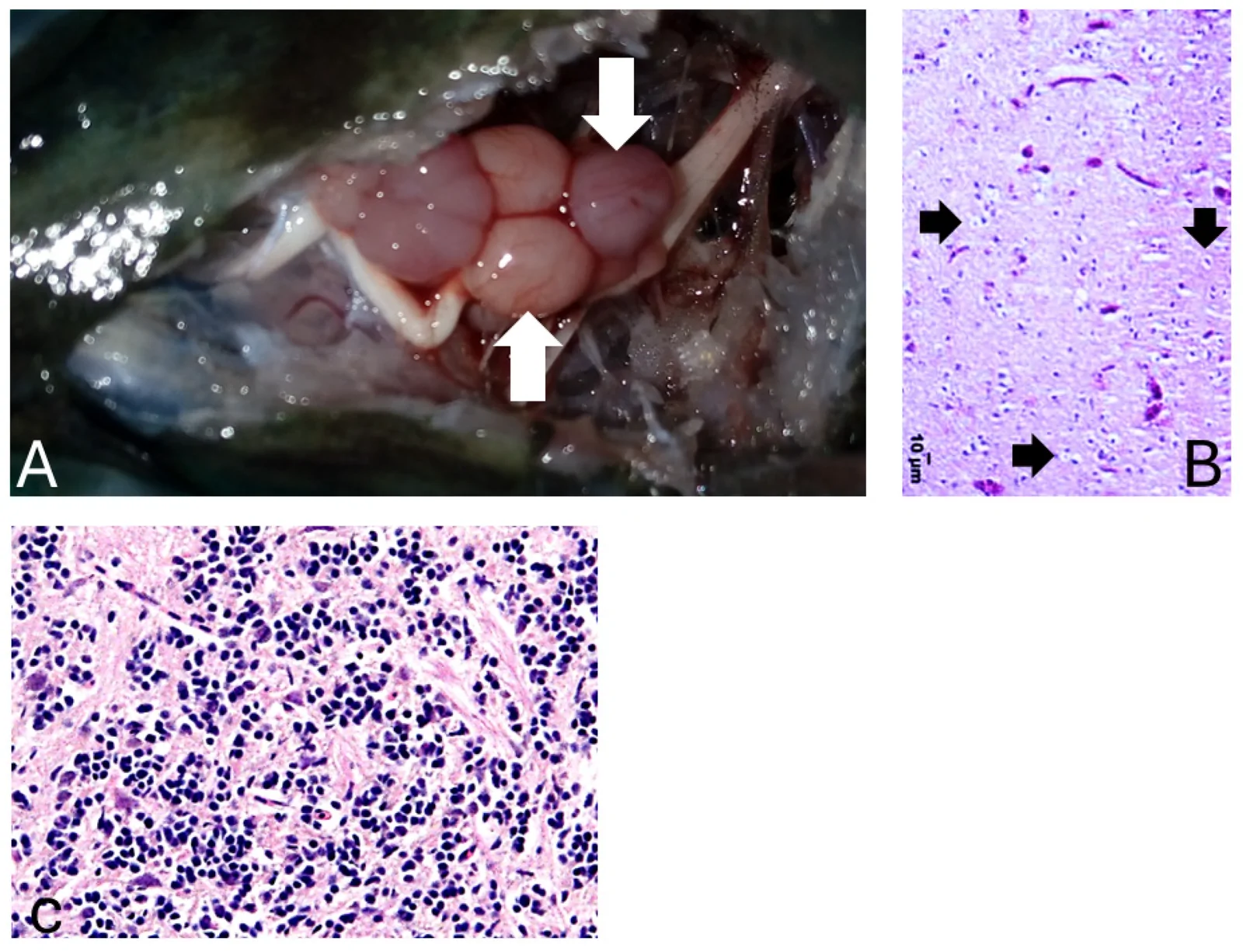
Red-spotted grouper nervous necrosis virus infection in Asian seabass. (**A**) Grossly congested and swollen brain (white arrows). (**B**) Histological sections showing vacuolation in the brain (arrows). (**C**) Control brain section. H&E stained.

Naturally infected Asian seabass exhibiting clinical signs (Figure 1) consistent with RGNNV infection were collected from sea cage farms in Karwar, Karnataka, India [15]. The presence of RGNNV was confirmed by histology and RT-PCR. Amplicons were subjected to Sanger dideoxy sequencing using ABI 3730xl DNA analyser at SciGenom Labs Pvt. Ltd., Kochi, India. The PCR products were sequenced to confirm the identity of the virus as the RGNNV genotype. Total RNA was extracted from the brain tissue of diseased fish using TRIzol Reagent (Thermo Fisher Scientific, Waltham, MA, USA) according to the manufacturer’s protocol. The integrity and purity of total RNA was assessed using a spectrophotometer, BioPhotometer plus (Eppendorf, Hamburg, Germany) and a NanoDrop spectrophotometer (Thermo Fisher Scientific, Waltham, MA, USA) including gel electrophoresis. The extracted total RNA samples were stored at −80 °C for further use.

### 2.2. Designing of LAMP Primers

**Figure 2:**
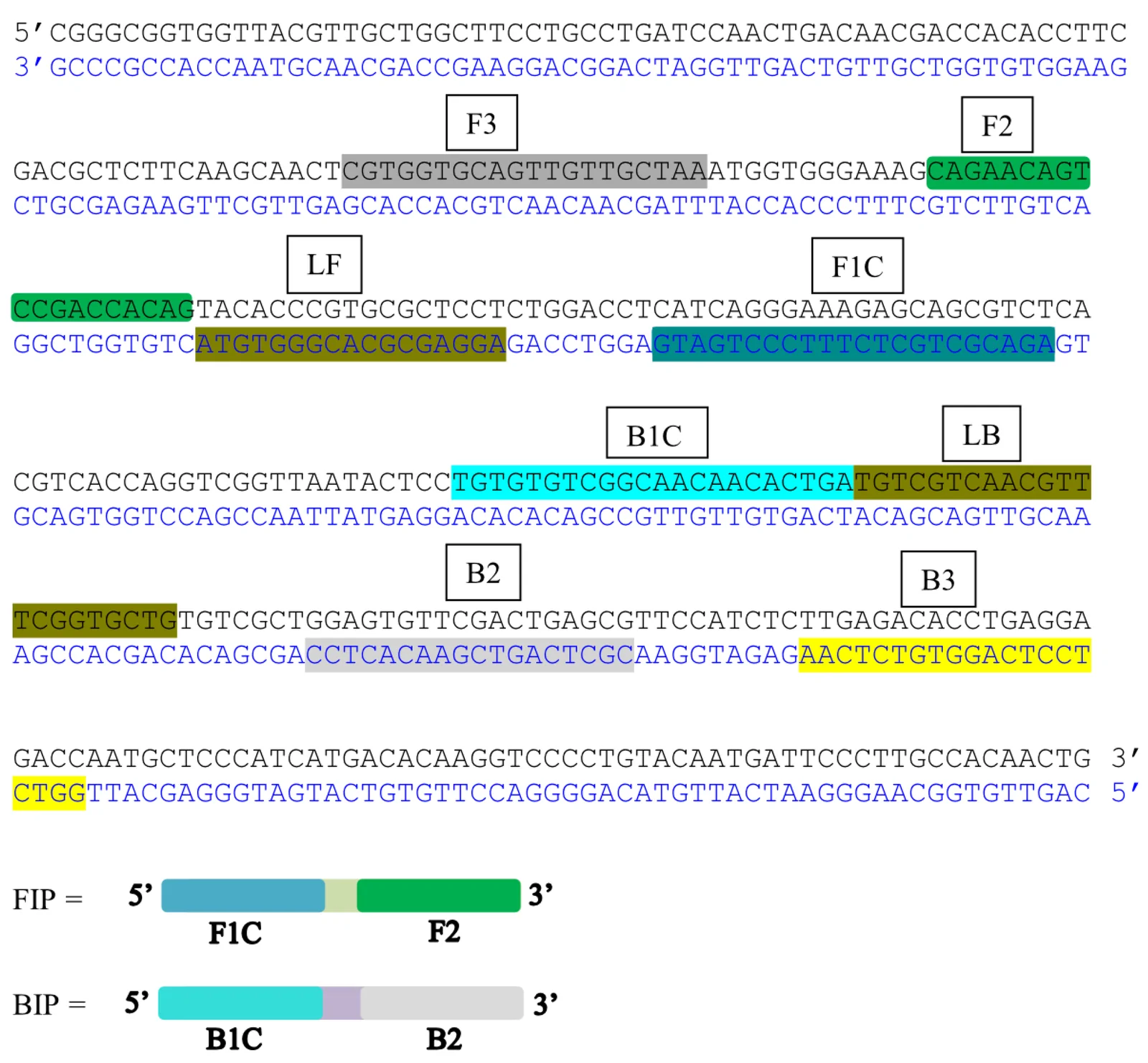
Nucleotide sequence of red-spotted grouper nervous necrosis virus used for the inner and outer primers. Primer Sequences used for primer design are highlighted in a different colour. Two outer (F3 and B3), two inner (FIC and BIC), and two loop primers (LF and LB) are designated by boxes. The forward inner primer (FIP) comprises the F1c and F2 regions, and the backward inner primer (BIP) comprises the B1C and B2 regions, each joined by a non-hybridising poly-T (ttttt) linker.

The primers were designed from highly conserved regions of RNA2 coat protein gene of the RGNNV genotypes using the online Primer Explorer V5 software https://primerexplorer.jp/e/ (accessed on 12 August 2019). The target region was selected based on the RGNNV RNA2 coat-protein gene sequence (GenBank accession no. MG202137.1). A set of six specific primers, two outer (F3 and B3), two inner (FIP and BIP) and two loop primers (LF and LB) were designed according to the guidelines provided. Additionally, the specificity of designed primers was evaluated in silico using NCBI BLASTN version 2.17.0 against the NCBI non-redundant database, applying an E-value threshold of 1 × 10^−3^. No significant homology was identified with non-betanodavirus sequences, confirming predicted specificity. The specific designed primers used for the amplification of RNA2 gene are given in Table 1. The primer sequences and their respective binding sites are shown in Figure 2.

### 2.3. Optimisation of the Single-Tube LAMP Assay

The RT-LAMP reaction was optimised at different reaction temperatures of 60, 61, 62, 63, 64, and 65 °C and different MgSO_4_ concentrations of 2, 4, 6, and 8 mM, with dNTPs concentrations varying from 0.8 to 2.0 mM each and reaction times from 30 to 60 min. The primer concentrations were similarly optimised over the ranges of 0.8–2.4 µM each for the inner primers (FIP/BIP), 0.1–0.4 µM each for the outer primers (F3/B3) and 0.2–0.8 µM each for the loop primers (LF/LB). The reaction parameters were optimised sequentially in the following order: (i) reaction temperature, (ii) MgSO_4_ concentration, (iii) dNTP concentration, (iv) inner primer (FIP/BIP) concentration, (v) outer primer (F3/B3) concentration, (vi) loop primer (LF/LB) concentration, and finally (vii) reaction time. Before the first optimisation step, all parameters were set at the working values commonly used for standard LAMP reactions: 65 °C, 6 mM MgSO_4_, 1.4 mM dNTPs (each), 1.6 µM FIP/BIP (each), 0.2 µM F3/B3 (each), 0.4 µM LF/LB (each), and a reaction time of 60 min [18,19]. Once a parameter was optimised, the selected value was carried forward and held constant for subsequent steps. Optimal conditions were determined based on the intensity of the SYBR Green I fluorescence signal, assessed visually by the naked eye or under a UV illumination at 365 nm together with the absence of fluorescence signal in the negative control. The details of the assay optimisation are provided in Supplementary Tables S1 and S2. All parameters were tested to optimise the LAMP reaction for the specific and rapid amplification of RGNNV. For the single-tube RT-LAMP, the reaction mixture was prepared by adding 5 µL of the total RNA to 12.5 µL 2X isothermal amplification buffer [40 mM Tris-HCl, 20 mM (NH_4_)_2_SO_4_, 100 mM KCl, 4 mM MgSO_4_, 0.2% Tween 20, dNTP Mix (1.4 mM each)], 5.75 µL of six primers mix [FIP and BIP (1.6 µM each), F3 and B3 (0.3 µM each), LF and LB (0.4 µM each)], 2 µL of enzyme mix [AMV RT (8000 U/mL) and Bst DNA polymerase (15,000 U/mL), New England Biolabs]. Nuclease free water was added to make up the final volume to 25 µL. The reaction was carried out in a dry block heater at 65 °C for 60 min, and the reaction was stopped by heating at 80 °C for 2 min. Afterwards, the reaction tube was opened carefully, and 1 µL of SYBR Green I (Invitrogen) was added (diluted 1:100). The tube was then tightly closed and sealed with parafilm. Then, the tubes were gently tapped so that SYBR Green I mixed thoroughly with the reaction product. Results were interpreted visually under a UV illuminator (Bio-Rad Laboratories, Hercules, CA, USA) at 365 nm and in ambient day light. A positive result was defined as the development of bright green fluorescence compared to a no-template negative control, which remained orange. A known positive RNA control was included in every reaction batch to confirm assay performance.

### 2.4. In Vitro RNA Transcription as a Positive Test Control

RNA2 coat protein gene of RGNNV originated from Asian seabass was cloned into a pJET 1.2 blunt-cloning vector (Thermo Fisher Scientific). The cloned gene was amplified using a forward primer incorporating a T7 RNA polymerase promotor sequence. The insert was confirmed by sequencing. The purified PCR products were used as templates. In vitro RNA transcription was carried out using T7 RNA polymerase (Thermo Fisher Scientific) according to the manufacturer’s protocol. The in vitro synthesis was treated with RNase-free DNase I (NEB) and then purified by phenol:chloroform:isoamyl alcohol extraction followed by ethanol precipitation [27]. The precipitated RNA was dissolved in nuclease-free water, quantified and stored in aliquots at −80 °C until further use.

### 2.5. Sensitivity of LAMP Assay

To determine the limit of detection (LOD), in vitro-transcribed RNA was serially diluted 10-fold (5, 50, 500 and 5000 copies/reaction) in nuclease-free water. Copy numbers were calculated from RNA concentration (ng/µL) measured spectrophotometrically, using the formula: copies/µL = [ng/µL × 6.022 × 10^23^]/[transcript length (nt) × 340 g/mol/nt × 10^9^]. All dilutions were tested in triplicate alongside a no-template negative control. The LOD was defined as the lowest concentration yielding a positive result in all three replicates. For comparative validation, the same dilution series was tested in parallel using the previously published RT-PCR method [15].

### 2.6. Evaluation and Specificity of LAMP Assay

The diagnostic performance of the RT-LAMP assay was evaluated in total RNA extracted from RGNNV-confirmed tissues collected from 30 Asian seabass and 30 cobias. In addition, total RNA extracted from 30 uninfected Asian seabass and 30 uninfected cobia was included as negative controls. The RGNNV infected fishes were collected from different places in Kerala (5 each of infected and uninfected Asian seabass), Karnataka (10 each of infected and uninfected Asian seabass) and Tamil Nadu (15 each of infected and uninfected Asian seabass and 30 each of infected and uninfected Cobia), India. All positive samples had been confirmed by histopathology, RT-PCR, and Sanger sequencing as described in Section 2.1 prior to inclusion. Briefly, total RNA (1 µg) was used to synthesize cDNA using M-MULV reverse transcriptase (NEB). The PCR reaction had a final volume of 20 µL, which contained 5 µL of 1:10-fold-diluted cDNA, 10 μM of each primer (VNN1_F264 3′-GTTGACGCAACCATCGTCC-5′ and VNN1_R1376 3′-CGGAGCTAACGGTAACCCAA-5′), Phusion™ High-Fidelity PCR Kit (New England Biolabs, Ipswich, MA, USA), and sterile distilled water. The PCR conditions were initial denaturation at 98 °C for 30 s, followed by 35 cycles of denaturation at 98 °C for 10 s, annealing at 59 °C for 30 s, and extension at 72 °C for 30 s. The final extension was performed at 72 °C for 5 min.

Clinical sensitivity and specificity were calculated relative to RT-PCR as the reference standard. Additionally, cross-reactivity of the RT-LAMP assay was assessed in total RNA from tilapia lake virus infected brain tissues of Nile tilapia (*Oreochromis niloticus*) and DNA from cyprinid herpesvirus-2 infected kidney tissues of ornamental goldfish (*Carassius auratus*).

### 2.7. Statistical Analysis

Sensitivity and specificity were calculated as the proportion of RT-PCR positive and negative samples correctly identified by the RT-LAMP assay. Positive predictive value and negative predictive value were calculated as the proportions of positive and negative RT-LAMP results. To assess the precision of the estimated detection parameters, exact 95% confidence intervals (CIs) were calculated using the Clopper–Pearson method. Statistical analyses were performed using IBM SPSS 28.0. A two-sided *p* value of <0.05 was considered statistically significant.

## 3. Results

### 3.1. Optimisation of Reaction

The LAMP assay was optimised to amplify the RGNNV RNA2 coat protein gene using FIP, BIP, F3, B3, LF, and LB primers by incubation at 65 °C for 60 min. The amplified product showed a ladder-like pattern on the gel, while no amplification was detected in the negative controls. Under the conditions tested, the strongest specific fluorescence was observed in the positive control, with no detectable signal in the negative control at 65 °C for 60 min with 4 mM MgSO_4_, 1.4 mM dNTPs (each), and inner, outer, loop primer concentrations of 1.6 µM, 0.3 µM, and 0.4 µM, respectively. These values were therefore adopted for all subsequent reactions.

**Figure 3:**
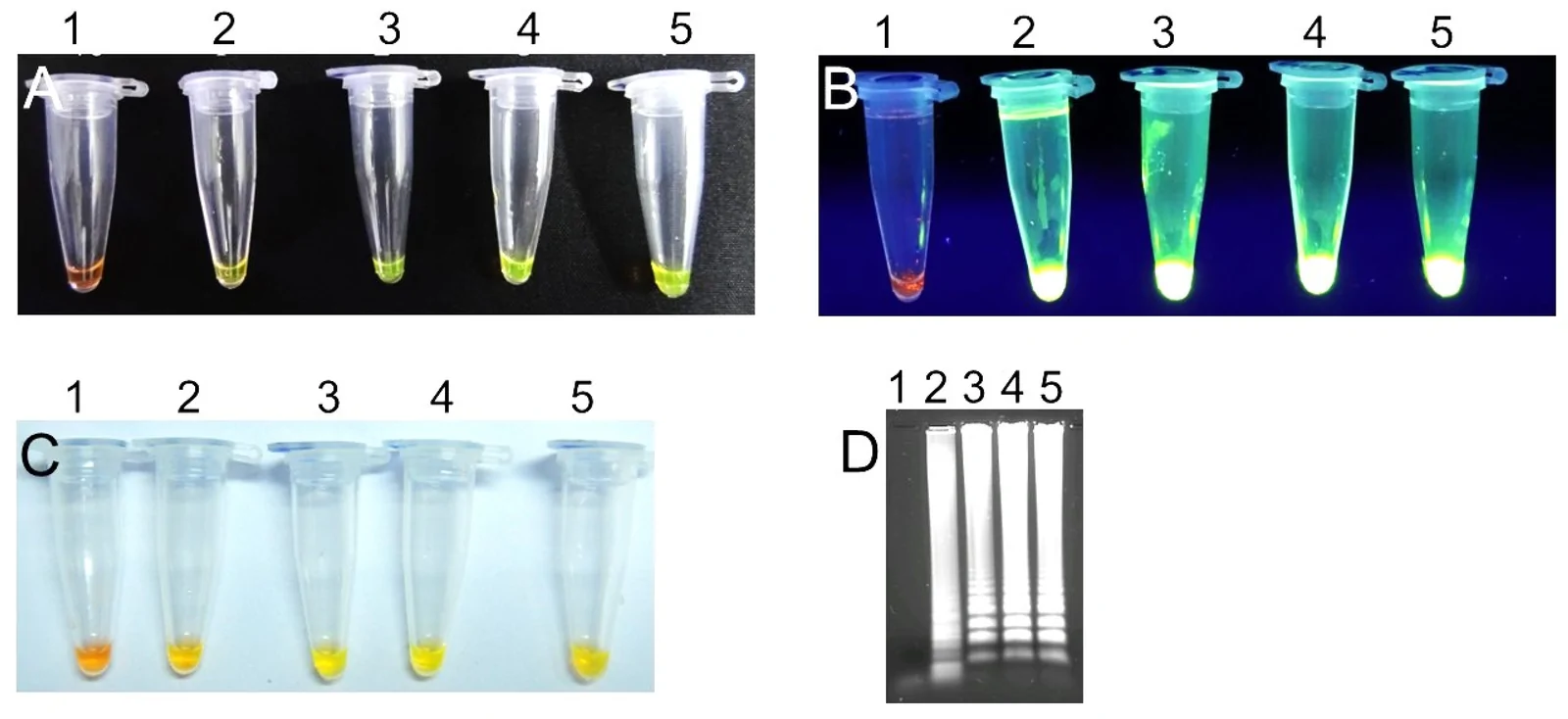
Detection sensitivity of RT-LAMP assay by addition of SYBR green I. (**A**) Observation under natural light with black background, (**B**) observation under UV light with black background, (**C**) observation under UV light with white background, (**D**) agarose gel electrophoresis and visualization under UV transilluminator (Bio-Rad Laboratories, Hercules, CA, USA). Tube 1: negative control, 1: tube 2: 5 copies, tube 3: 50 copies, tube 4: 500 copies and tube 5: 5000 copies of in vitro-synthesized red-spotted grouper nervous necrosis virus RNA 2 coat protein RNA. Positive reactions yielded a bright green colour upon addition of SYBR Green I. Negative control remained unchanged (orange colour).

Positive RT-LAMP reactions yielded a bright green colour upon addition of SYBR Green I (1:100 dilution), clearly distinguishable from the orange colour of negative controls under both ambient daylight and UV illumination (365 nm) regardless of background (Figure 3). Negative controls and no-template controls remained orange under all observation conditions. This colour change was also observable with the naked eye under normal light. Positive reactions emitted green fluorescence when exposed to UV light with a white background, while negative controls were unchanged (orange colour).

### 3.2. Sensitivity of the Assay

The analytical sensitivity of the RT-LAMP assay was determined using a 10-fold serial dilution series of in vitro-transcribed RGNNV RNA2. The assay consistently detected five copies/reaction, establishing a limit of detection (LOD) of five copies/reaction (Figure 3). Under identical conditions, the conventional RT-PCR assay yielded a LOD of 100 copies/reaction [15], demonstrating a 20-fold higher analytical sensitivity of developed RT-LAMP assay.

### 3.3. Evaluation and Specificity of the Assay

**Figure 4:**
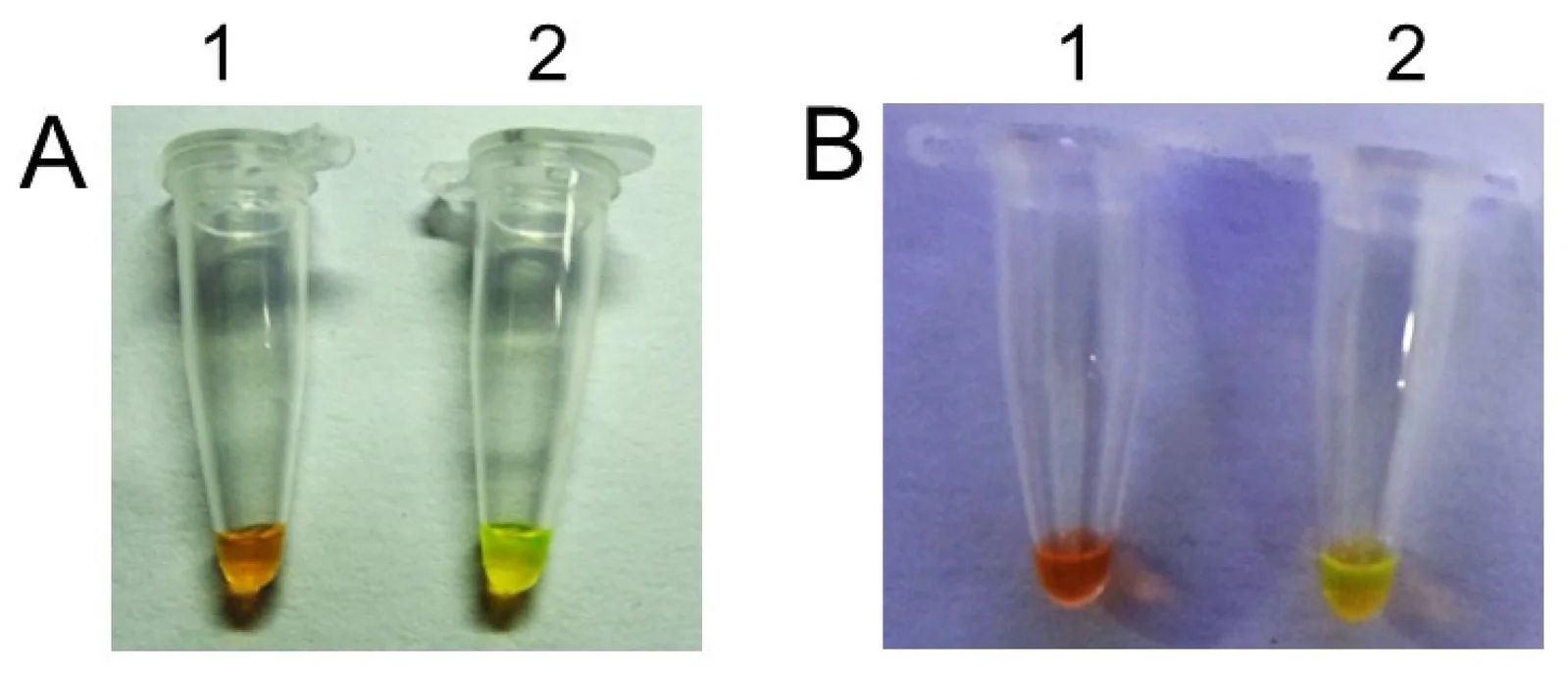
Detection of red-spotted grouper nervous necrosis virus in total RNA isolated from tissues by RT-LAMP assay. (**A**) brain tissue of cobia, (**B**) brain tissue of Asian seabass. The positive samples exhibited a fluorescent green colour under natural light following the addition of SYBR Green I dye. Negative control remained unchanged (orange colour). Tube 1: Total RNA sample from uninfected tissue (negative control); tube 2: total RNA sample from RGNNV infected tissue.

The specificity of the assay was successfully evaluated in clinical samples from two fish species (Figure 4). The RT-LAMP assay correctly identified all 30 reverse transcription-PCR-confirmed RGNNV-positive samples from Asian seabass and cobia, yielding a clinical sensitivity of 100% within the test sample set. Likewise, all 30 uninfected control samples per species were negative by RT-LAMP, consistent with RT-PCR results. The assay did not show any cross-reaction with tilapia lake virus and cyprinid herpesvirus-2, uninfected control tissues and negative templates. The corresponding exact 95% confidence intervals were 88.4–100% for both sensitivity and specificity.

## 4. Discussion

The LAMP technique has attracted considerable attention as a rapid and highly sensitive diagnostic tool for fish pathogens. The initial use of LAMP for the detection of aquatic pathogens was reported for edwardsiellosis [28]. *Edwardsiella tarda* isolated from a diseased Japanese flounder, *Paralichthys olivaceus,* was detected using primers targeting the haemolysin gene. The LAMP-based detection was more sensitive than PCR, where it was capable of detecting 10 colony-forming units (CFUs) as compared to 10^3^ CFUs detected using PCR. Since then, LAMP and its variant RT-LAMP have been applied for the detection of various viral and bacterial pathogens in fish. Although a few studies reported RT-LAMP assays for detecting RGNNV caused by betanodaviruses originated from China and Japan [23,26], most of these assays were restricted to a single host species. The present study addresses this gap by developing a single-tube RT-LAMP assay targeting a conserved region of the RGNNV RNA2 coat-protein gene and evaluating it in more than one commercially important host species.

Sung and Lu [22] developed an RT-LAMP assay that could detect the nervous necrosis virus in groupers within two hours. This method showed greater sensitivity than one-step and nested PCR. Similarly, Xu et al. [23] used LAMP for the detection of RGNNV Chinese strain in *Trachinotus ovatus*. They reported a sensitivity 100-fold higher than nested PCR. Suebsing et al. [24] developed an RT-LAMP assay for the rapid, sensitive, and cost-effective detection of nervous necrosis virus (NNV) in olive flounder (*Paralichthys olivaceus*). A set of six specific primers was designed to target the RNA2 gene encoding the coat protein of Korean NNV strains. This RT-LAMP successfully detected NNV after 30 min at 65 °C. When the sensitivities among RT-LAMP, RT-PCR, and nested PCR were compared, the RT-LAMP was demonstrated to be able to detect the RNA template at 2.58 × 10^−2^ TCID_50_/mL, whereas the RT-PCR and nested PCR were only able to detect the RNA template at 2.58 × 10^2^ TCID_50_/mL and 2.58 TCID_50_/mL, respectively.

The findings of these results showed that the sensitivity of the RT-LAMP assay was higher than that of conventional RT-PCR methods. In specificity tests, two NNV genotypes (SJNNV and RGNNV) were successfully detected in olive flounder, while no other fish viruses were amplified, indicating that the RT-LAMP assay is only specific to NNV. Similarly, Hwang et al. [25] also developed LAMP for the identification of NNV infection in sevenband grouper, *Epinephelus septemfasciatus*. The sensitivity of LAMP for the detection of NNV was 10-fold higher than that of PCR. Mekata et al. [26] further developed real-time RT-LAMP using the GNNV RdRP gene (GenBank: AY324869) for the detection of the Japanese RGNNV strain in grouper fish. However, this assay required a Loopamp real-time turbidimeter to detect amplified products by measuring optical density at 650 nm. This reliance on specialized instrumentation increases the assay’s overall cost and limits its portability for field-based applications.

Despite these advancements, assays have mostly been restricted to certain viral genotypes or fish species. The present RT-LAMP assay was designed to target a highly conserved region within the T4 variable domain of the RNA2 coat protein gene of RGNNV (GenBank: MG202137.1), a region previously shown to be phylogenetically informative and conserved across Indian RGNNV isolates [15]. We developed a set of six primers to target eight conserved regions of the RNA2 coat protein gene. It included loop primers that enhanced amplification efficiency and sensitivity. The developed RT-LAMP assay demonstrated an analytical LOD of five RGNNV RNA copies per reaction. In comparison, Mekata et al. [26] reported a real-time RT-LAMP assay capable of detecting as few as 4 × 10^−11^ copies of RGNNV RNA per µL, whereas Hwang et al. [25] reported a detection limit of 2.58 × 10^−2^ TCID_50_/mL of NNV. Similarly, Xu et al. [23] reported that their RT-LAMP assay was approximately 100-fold more sensitive than nested PCR. However, direct comparison of the analytical sensitivities among these assays is difficult because the detection limits were determined using different units (copies/µL, TCID_50_/mL and relative to nested PCR) and were calculated in different target materials, assay formats, and experimental conditions.

Our developed RT-LAMP assay provided a rapid (≤60 min), sensitive (LOD: five copies/reaction) and specific diagnostic platform for the detection of RGNNV. In addition, the assay demonstrated complete agreement with the RT-PCR assay for the clinical samples evaluated and provided visual detection within 60 min using only a simple heating device. Unlike several previous studies that focused on a single host species, the present assay was successfully validated using clinical samples obtained from both Asian seabass and cobia, demonstrating its applicability across multiple important marine fish species.

The RT-LAMP assay requires only basic laboratory equipment, making it simpler and less expensive to set up than conventional RT-PCR. It also gives results quickly. Although a formal cost analysis was not performed in the present study, the absence of PCR thermal cycler equipment reduces assay time and the overall cost of diagnosis for routine surveillance in hatcheries and diagnostic laboratories.

Nevertheless, the present study has certain limitations. The assay was designed to detect RGNNV genotype. The diagnostic performance was tested in two fish species, and specificity was assessed against two viral pathogens. The ability of the present assay was not evaluated with SJNNV, TPNNV, and BFNNV genotypes. In addition, the assay was validated under laboratory conditions using purified RNA and clinical samples, and its performance under true farm-side conditions was not assessed. Some factors such as sample quality, RNA degradation, and improper sample handling may influence diagnostic performance and occasionally result in false-negative reactions. Furthermore, the long-term stability of RT-LAMP reagents under field conditions was not evaluated in this study and requires further investigation. Future studies are needed to evaluate the assay using a larger number of clinical samples and susceptible fish species, as well as a broader panel of fish viruses and geographically diverse betanodavirus isolates.

## 5. Conclusions

We developed a single-tube RT-LAMP assay for the rapid, cost-effective, and specific detection of RGNNV in fish tissue samples. Its compatibility with simple heating equipment and visual readout makes it suitable for deployment in resource-limited aquaculture diagnostic laboratories. It can be effectively implemented in modestly equipped laboratories, aquaculture farm sites, mobile diagnostic units, broodstock facilities, quarantine facilities, and for the screening of fingerlings and juveniles prior to stocking in ponds or cages. This assay would be useful for the aquaculture industry in the timely detection of RGNNV, enabling the development of suitable strategies to control the disease and its occurrence and transmission.

## Figures and Tables

**Table 1:** Primers used for the single-tube RT-LAMP assay to detect red-spotted grouper nervous necrosis virus. The table presents the sequences of six primers designed from conserved regions of the RNA2 gene encoding the coat protein. Lowercase ‘ttttt’ denotes a non-hybridising poly-T linker between F2 and F1c regions of inner primers FIP and BIP.

Primer Name	Sequence 5′–3′	Length (mer)
F3	CGTGGTGCAGTTGTTGCTAA	20
B3	GGTCTCCTCAGGTGTCTCAA	20
FIP	AGACGCTGCTCTTTCCCTGATGtttttCAGAACAGTCCGACCACAG	46
BIP	TGTGTGTCGGCAACAACACTGAtttttCGCTCAGTCGAACACTCC	45
LF	AGGAGCGCACGGGTGTA	17
LB	TGTCGTCAACGTTTCGGTGCTG	22

## Data Availability

All the data are provided within the article. Further inquiries can be directed to the corresponding authors.

## Supplementary Materials

The following supporting information can be downloaded at: https://www.mdpi.com/article/10.3390/v18080827/s1, Table S1: Factors and levels evaluated in the RT-LAMP optimisation; Table S2: Optimisation of the RT-LAMP reaction conditions.

## Abbreviations

BFNNV	Barfin flounder nervous necrosis virus
RGNNV	Ted-spotted grouper nervous necrosis virus
RT-LAMP	Reverse Transcription–Loop-Mediated Isothermal Amplification
PCR	Polymerase chain reaction
TPNNV	Tiger puffer nervous necrosis virus
VER	Viral encephalopathy and retinopathy
VNN	Viral nervous necrosis
