# Supplementary material for: Single-Tube Reverse Transcription–Loop-Mediated Isothermal Amplification Assay for Rapid Detection of Red-Spotted Grouper Nervous Necrosis Virus in Fish Species
Source: Viruses. 2026 Jul 27;18(8):827. doi: 10.3390/v18080827 (PMC13517385; doi:10.3390/v18080827)
Supplement: Supplementary file 1 [file viruses-18-00827-s001.zip › viruses-4426638-supplementary.pdf]

viruses

MDPI

Supplementary Data

Design of Experiments for RT-LAMP Optimisation

The method was implemented as a sequential, one-factor-at-a-time univariate optimization. Each factor was varied across its levels while the remaining factors were held at their working/selected values. Optimisation began with the reaction temperature and proceeded in the order indicated in the "Optimisation step" column of Table S1, namely: reaction temperature (1st), $MgSO_4$ concentration (2nd), dNTP concentration (3rd), inner primer (FIP/BIP) concentration (4th), outer primer (F3/B3) concentration (5th), loop primer (LF/LB) concentration (6th), and reaction time (7th). Reaction time was evaluated last, with all other factors fixed at their selected optimum values. Before the first optimisation step, every factor was set at the initial working value listed in Table S1; these correspond to the concentrations and conditions commonly used for standard LAMP reactions (Notomi et al., 2000; Tomita et al., 2008). Once a factor had been optimised, its selected value was carried forward and held constant during all subsequent steps.

Table S1. Factors and levels evaluated in the RT-LAMP optimisation.

Code	Optimisation step	Factor	Unit	Initial working value	Levels tested	No. of levels	Selected optimum
A	1st	Reaction temperature	°C	65	60, 61, 62, 63, 64, 65	6	65
B	2nd	$MgSO_4$ concentration	mM	6	2, 4, 6, 8	4	4
C	3rd	dNTP (each)	mM	1.4	0.8, 1.2, 1.4, 1.6, 2.0	5	1.4
D	7th	Reaction time	min	60	30, 40, 50, 60	4	60
E	4th	Inner primers (FIP/BIP, each)	µM	1.6	0.8, 1.2, 1.6, 2.0, 2.4	5	1.6
F	5th	Outer primers (F3/B3, each)	µM	0.2	0.1, 0.2, 0.3, 0.4	4	0.3
G	6th	Loop primers (LF/LB, each)	µM	0.4	0.2, 0.4, 0.6, 0.8	4	0.4

Table S2. Optimization of the RT-LAMP reaction conditions. Each parameter was optimized sequentially (one variable at a time, with the remaining components held at their working values). Optimal conditions were selected on the basis of SYBR Green I fluorescence signal intensity in the positive control and the absence of signal in the negative control.

**Parameter**	**Range Tested**	**Optimal Value selected**	**Basis of selection**
$MgSO_4$	2-8 mM	4 mM	determined based on the intensity of the SYBR Green I fluorescence signal in the positive control and the absence of signal in the negative control
dNTPs (each)	0.8–2.0 mM	1.4 mM	
Inner primers (FIP/BIP)	0.8–2.4 µM	1.6 µM	
Outer primers (F3/B3)	0.1–0.4 µM	0.3 µM	
Loop primers (LF/LB)	0.2–0.8 µM	0.4 µM	
Incubation temperature	60–65 °C	65 °C	
Reaction time	30–60 min	60 min	

Under the conditions tested, the strongest specific fluorescence was observed in the positive control, with no detectable signal in the negative control at 65 °C for 60 minutes with 4 mM $MgSO_4$, 1.4 mM dNTPs (each), and inner, outer, loop primer concentrations of 1.6 µM, 0.3 µM, and 0.4 µM, respectively.

Disclaimer/Publisher's Note: The statements, opinions and data contained in all publications are solely those of the individual author(s) and contributor(s) and not of MDPI and/or the editor(s). MDPI and/or the editor(s) disclaim responsibility for any injury to people or property resulting from any ideas, methods, instructions or products referred to in the content.

https://doi.org/10.3390/v18080827
